# Data on gut metagenomes of the patients with *Helicobacter pylori* infection before and after the antibiotic therapy

**DOI:** 10.1016/j.dib.2017.01.007

**Published:** 2017-01-17

**Authors:** Oksana E. Glushchenko, Andrei E. Samoilov, Evgenii I. Olekhnovich, Boris A. Kovarsky, Alexander V. Tyakht, Alexander V. Pavlenko, Vlad V. Babenko, Andrei K. Larin, Elena S. Kostryukova, Maja V. Malakhova, Elena N. Ilina, Rustem A. Abdulkhakov, Dilyara I. Safina, Tatiana V. Grigoryeva, Sayar R. Abdulkhakov, Vadim M. Govorun

**Affiliations:** a Federal Research and Clinical Centre of Physical-Chemical Medicine, Malaya Pirogovskaya 1a, Moscow 119435, Russia; b Moscow Institute of Physics and Technology, Institutskiy per. 9, Dolgoprudny, Moscow Region 141700, Russia; c Kazan State Medical University, Butlerova 49, Kazan 420012, Russia; d Kazan Federal University, Kremlyovskaya 18, Kazan 420008, Russia

**Keywords:** Gut microbiota, Metagenome, Antibiotic therapy, *Helicobacter pylori*

## Abstract

Antibiotic therapy can lead to the disruption of gut microbiota community with possible negative outcomes for human health. One of the diseases for which the treatment scheme commonly included antibiotic intake is *Helicobacter pylori* infection. The changes in taxonomic and functional composition of microbiota in patients can be assessed using “shotgun” metagenomic sequencing. Ten stool samples were collected from 4 patients with *Helicobacter pylori* infection before and directly after the *H. pylori* eradication course. Additionally, for two of the subjects, the samples were collected 1 month after the end of the treatment. The samples were subject to “shotgun” (whole-genome) metagenomic sequencing using Illumina HiSeq platform. The reads are deposited in the ENA (project ID: PRJEB18265).

**Specifications Table**

**Table:** 

Subject area	Biology
More specific subject area	Bacterial metagenomics
Type of data	Text files: sequences
How data was acquired	DNA sequencing using Illumina HiSeq 2500 platform
Data format	Raw
Experimental factors	DNA extracted from stool samples
Experimental features	Two micrograms of total DNA per sample were used to create barcoded paired-end sequencing libraries. “Shotgun” sequencing was performed using Illumina HiSeq 2500 platform according to the recommendations of the manufacturer.
Data source location	Kazan, Russian Federation
Data accessibility	The data is deposited in the European Nucleotide Archive (project ID: PRJEB18265, URL: http://www.ebi.ac.uk/ena/data/view/PRJEB18265).

**Value of the data**

• The data can be used to assess the antibiotic-induced gut dysbiosis in a cultivation-independent manner via the changes in the relative abundance of microbial taxa and genes.
• Using the metagenomic datasets corresponding to three consequent time points - before, immediately after and 1 month after the end of the treatment - it is possible to analyze the temporal dynamics and resilience of gut microbial ecosystem.
• By calculating the relative abundance of the genes conferring antibiotic resistance from the data, researchers can evaluate the resistome of gut microbiota and its changes during and after the antibiotic intake.
• Identification of specific changes in gut microbiota composition as the result of *H. pylori* eradication using the presented data opens the way to designing new approaches to the alleviation of the side effects of the treatment.

## 1 Data

The presented dataset contains 10 “shotgun” human gut metagenomes assessed from stool samples from the patients with *Helicobacter pylori* infection. The total read length for the dataset is 87.6 Gbp (the metagenomes contain 34.1±13.6 mln of reads per sample, mean±s.d.). Details about the dataset are shown in Table 1.

## 2 Experimental design, materials and methods

### 2.1 Cohorts assembly

The study was approved by the Local ethics committee of the Kazan (Volga region) Federal University. Each patient signed an informed consent before the start of the study. The patients were enrolled in University Hospital of Kazan Federal University (former Republican Clinical Hospital #2 of the Ministry of Healthcare of Republic of Tatarstan).

### 2.2 Patients inclusion and exclusion criteria

General inclusion criteria: males and females, age 18–75 years with performed upper GI endoscopy and *H. pylori*-positive status 1 month prior to the inclusion into the study (according to any of the *H. pylori* detection methods). Exclusion criteria: malignancies; inability of the patient to follow the study procedures; concomitant diseases possibly influencing the gut microbiota composition including inflammatory bowel diseases, malabsorption (due to small intestine diseases or pancreatic insufficiency) and other diseases; abdominal surgery (excluding appendectomy); intake of medications (immunomodulators, corticosteroids, non-steroidal anti-inflammatory drugs, antibiotics, pre- and probiotics - for 3 months prior to the inclusion; proton pump inhibitors, bismuth-containing medications - for 2 weeks prior to inclusion); alcohol or drug abuse; decompensated chronic diseases; infectious diseases including HIV, viral hepatitis, tuberculosis and others; presence of diarrhea (bowel movement frequency >3 times a day) for at least 3 consequent days during the last month; pregnancy or breastfeeding.

### 2.3 Sample collection and metagenomic sequencing

Stool samples were collected from the subjects, stored and subject to DNA extraction as described before [1]. Barcoded paired-end libraries were created from 2 μg of total DNA for each sample according to the manufacturer׳s recommendations using NEBNext DNA Library Prep Master Mix Set for Illumina (New England Biolabs, USA) and NEBNext Multiplex Oligos for Illumina (96 Index Primers) (New England Biolabs, USA) kits. “Shotgun” metagenomic sequencing was performed on HiSeq 2500 platform (Illumina, USA) according to the manufacturer׳s recommendations using the following reagent kits: HiSeq Rapid PE Cluster Kit v2, HiSeq Rapid SBS Kit v2 (500 cycles), HiSeq Rapid PE FlowCell v2. The resulting read length was 250 bp.

## Figures and Tables

**Table 1:** **Description of the metagenomic datasets**. In “Time point” column, the numbers show the point of sample collection: 1 - before the treatment, 2 - immediately after the end of the treatment, 3 - one month after the end of the treatment. Abbreviations: ENA – European Nucleotide Archive, BMI - body-mass index. The patients for whom the samples were collected at three time points include P3 and P4.

**Sample ID**	**ENA sample number**	**ENA experiment number**	**Time point**	**Internal patient ID**	**Gender**	**Age**	**BMI**
Hp_21_S2	ERS1462079	ERX1813428	1	P1	M	31	28.07
Hp_22_S23	ERS1462080	ERX1813429	2				
Hp_23_S25	ERS1462081	ERX1813430	1	P2	F	63	27.39
Hp_24_S11	ERS1462082	ERX1813431	2				
Hp_5_S19	ERS1462083	ERX1813432	1	P3	F	31	18.49
Hp_6_S28	ERS1462084	ERX1813433	2				
Hp_7_S14	ERS1462088	ERX1813437	3				
Hp_73_S10	ERS1462085	ERX1813434	1	P4	M	54	24.06
Hp_74_S4	ERS1462086	ERX1813435	2				
Hp_76_S8	ERS1462087	ERX1813436	3				
